# The path from trigeminal asymmetry to cognitive impairment: a behavioral and molecular study

**DOI:** 10.1038/s41598-021-82265-6

**Published:** 2021-02-26

**Authors:** Maria Paola Tramonti Fantozzi, Giulia Lazzarini, Vincenzo De Cicco, Angela Briganti, Serena Argento, Davide De Cicco, Massimo Barresi, Enrico Cataldo, Luca Bruschini, Paola d’Ascanio, Andrea Pirone, Carla Lenzi, Iacopo Vannozzi, Vincenzo Miragliotta, Ugo Faraguna, Diego Manzoni

**Affiliations:** 1 Department of Translational Research and of New Surgical and Medical Technologies, University of Pisa, Via San Zeno 31, 56127 Pisa, Italy; 2 Department of Veterinary Sciences, University of Pisa, Pisa, Italy; 3 Department of Neurosciences, Reproductive and Odontostomatological Sciences, University of Naples “Federico II”, Naples, Italy; 4 Department of Physics, University of Pisa, Pisa, Italy; 5 Department of Surgical, Medical, Molecular Pathology and CriticalCare Medicine, University of Pisa, Pisa, Italy; 6 Department of Developmental Neuroscience, IRCCS Fondazione Stella Maris, Pisa, Italy

**Keywords:** Cognitive neuroscience, Neural circuits, Sensory processing

## Abstract

Trigeminal input exerts acute and chronic effects on the brain, modulating cognitive functions. Here, new data from humans and animals suggest that these effects are caused by trigeminal influences on the Locus Coeruleus (LC). In humans subjects clenching with masseter asymmetric activity, occlusal correction improved cognition, alongside with reductions in pupil size and anisocoria, proxies of LC activity and asymmetry, respectively. Notably, reductions in pupil size at rest on the hypertonic side predicted cognitive improvements. In adult rats, a distal unilateral section of the trigeminal mandibular branch reduced, on the contralateral side, the expression of c-Fos (brainstem) and BDNF (brainstem, hippocampus, frontal cortex). This counterintuitive finding can be explained by the following model: teeth contact perception loss on the lesioned side results in an increased occlusal effort, which enhances afferent inputs from muscle spindles and posterior periodontal receptors, spared by the distal lesion. Such effort leads to a reduced engagement of the intact side, with a corresponding reduction in the afferent inputs to the LC and in c-Fos and BDNF gene expression. In conclusion, acute effects of malocclusion on performance seem mediated by the LC, which could also contribute to the chronic trophic dysfunction induced by loss of trigeminal input.

## Introduction

### Trigeminal input and brain functions

Chewing improves alertness^1,2^, speeds up cognitive processing^3^, shortens the reaction time (reducing the latencies of the corresponding event-related potentials^1,3–5^). Along with the improvement in cognitive tasks, it also increases cerebral blood oxygen-dependent signal^3^ and task-related pupil dilatation (mydriasis)^6^. These effects are rather specific, as they cannot be attributed to motor arousal only^5,6^. Furthermore, chewing-induced changes in cognitive performance and in task-related mydriasis are positively correlated, suggesting a common driving mechanism^6^.

Moreover, subjects showing an asymmetry in masseter electromyographic (EMG) activity during clenching (imbalance in trigeminal sensorimotor activity) improve cognitive performance and mydriasis at rest when the EMG asymmetry is corrected by an orthosis^7,8^.

The link between trigeminal input and cognition might contribute to the development of neurodegerative disorders. In humans, a good masticatory function helps to preserve brain functions^9^, reducing the risk of developing Alzheimer’s disease and other forms of dementia (AD)^10,11^. In aged mice, masticatory rehabilitation significantly improves spatial memory^12^.

Bilateral molar extraction or removal of teeth crowns in adult mutant mice overexpressing amyloid-β precursor protein (APP)^13^ or in adult/aged SAMP8 mice (a model of accelerated aging)^14,15^ results in memory impairment, with cell and dendritic spines loss at hippocampal level.^.^ Moreover, teeth crown resection enhances apoptosis markers and β-amyloid levels^16^ (one of the AD markers^17^) in Wistar rats, while reduces the expression of Brain-Derived Neurotrophic Factor (BDNF)^18^ in aged SAMP 8 mice. A reduction in the expression of tyrosine kinase receptor B, involved in some forms of synaptic plasticity, has been reported in Wistar rats^19^.

Severe deficits in spatial memory could be observed in Wistar rats^19^ as well as in aged SAMP8 mice^20^; in the latter preparation a reduced hippocampal activation during spatial tasks^21^ was also observed. The deficits described in SAMP8 mice worsened with animal age and time following teeth removal^14^ and were prevented by applying artificial crowns^21^. Moreover, adult and aged mices preserving a complete set of teeth and undergoing a soft diet^22–25^ showed similar impairments.

### Acute effects

Trigeminal signals are the most powerful^26^ cranial afferent in sustaining the activity of the Ascending Reticular Activating System (ARAS)^27,28^, which enhances arousal and performance during waking^29^.

In this perspective, an asymmetric trigeminal sensorimotor activity may induce, through the ARAS, an imbalance in cortical activation. This asymmetry would, in turn, lead to an impairment in cognitive function. There is indeed evidence that a cortical imbalance, obtained by a unilateral lesion, is more detrimental for cognitive functions as compared to a double symmetric lesion^30^. Unexpectedly, the cognitive deficits induced by a unilateral cortical lesion may disappear following a second, symmetric lesion^30^, thus indicating that the lesion-induced cortical imbalance and not the lesion itself is responsible for the initial deficits. Moreover, cognitive deficits caused by asymmetric cerebral lesions in humans are reduced by asymmetric sensory stimulations^31^, applied to correct the cortical imbalance.

Trigeminal pathways to ARAS implicated in cognitive control^32^ engage the noradrenergic Locus Coeruleus (LC) neurons^33^. LC activity, estimated by recording the pupil size^34–39^, is modified by the trigeminal input. The asymmetry in the EMG activity of the masseter muscles observed during clenching, triggering an imbalance in the afferent input both from muscle spindles and periodontal receptors^7,8^, is in fact positively correlated with an asymmetry in pupil size (anisocoria) at rest. Both the anisocoria and the EMG asymmetry disappear when a correcting bite is applied^7,8^.

Moreover, chewing-induced stimulation of cognitive performance develops in parallel with enhancement of task-related pupil dilatation (mydriasis)^6^, which is due to the concomitant phasic activation of LC neurons during task^40,41^, thus suggesting that chewing effects on cognitive performance are attributable to LC activation.

### Chronic effects

The LC could be also involved in the chronic, neurodegenerative effects observed following teeth removal^32^. This structure affects neurovascular coupling, maintains the integrity of blood brain barrier, feeds astrocyte metabolic activity and intracellular Ca^2+^ levels^32,42^, stimulates the phagocytosis of β-amyloid plaque by microglia^43,44^, regulates β-amyloid levels^45^ and inhibits the release of cytokines and chemokines, underlying the inflammatory reaction of microglia^46^. The degeneration of LC occurs very early in Parkinson’s and Alzheimer’s disease^47,48^ and it has been proposed that LC neurons can trigger neurodegenerative processes, which diffuse to other forebrain structures through noradrenergic fibres^49,50^.

LC development depends upon trigeminal sensory neurons, supplying LC with the Onecut transcription factors, fundamental for survival and differentiation of its neurons^51^. Masseter muscle fibres and spindles, teeth and vibrissae provide also neurotrophic factors^32,52^, which sustain the survival of LC neurons^53–55^ and whose action persists across adult age^54,56^. Loss of trigeminal afferent inputs may reduce the availability of neurotrophins for LC neurons, leading to their dysfunction/degeneration^32^ and, possibly, to reduced brainstem levels of norepinephrine-synthesizing enzymes dopamine-β-hydroxylase (DBH) and tyrosine hydroxylase (TH), whose expression is activity-dependent^57–59^.

### Aims of the study

The first aim (Aim I) of the study was to investigate whether the effects of the correction of an asymmetric trigeminal input upon cognitive performance are mediated by a shift in LC activity^7,8^, inferred by pupil size recording (De Cicco et al., 2014, 2016).

To address Aim I, we recruited a population of human subjects showing a significant asymmetry in the EMG activity of masseter muscles during clenching. In these subjects, an occlusal correction (using a bite) rescued the EMG activity balance during clenching. We verified whether bite wearing, in two different mandible positions (arches opened and closed), acutely affected cognitive performance, while monitoring pupil size and task-related mydriasis.

The second aim (Aim II) was to document whether a chronic trigeminal imbalance in adult Wistar rats may lead to molecular signs of LC asymmetry, by performing either a unilateral removal of the cusps of the molar teeth, or a unilateral distal section of the trigeminal mandibular branch. In particular, we investigated whether these sections asymmetrically affected the expression of norepinephrine-synthesizing enzymes in the brainstem and of the LC-dependent c-Fos and BDNF coding genes in LC target regions^60–62^.

## Results

### Aim I

#### EMG asymmetry and baseline evaluations within the population analysed

Baseline values of pupil size, task-related mydriasis, and performance parameters are given in Table 1 for all the four sessions of the preliminary study (see “Methods”).

**Table 1:** Mean ± SD of pupil size and performance parameters obtained in four different experiments (Trial 1–4) performed in 30 subjects.

N = 30	Trial 1	Trial 2	Trial 3	Trial 4	Average
Pupil size	3.82 ± 0.73	3.84 ± 0.76	3.84 ± 0.75	3.90 ± 0.79	3.85 ± 0.61
Mydriasis	1.46 ± 0.33	1.45 ± 0.34	1.47 ± 0.36	1.36 ± 0.36	1.44 ± 0.28
PI	1.74 ± 0.49	1.76 ± 0.53	1.75 ± 0.51	1.75 ± 0.55	1.75 ± 0.39
SR	12.98 ± 2.09	12.92 ± 2.08	12.96 ± 2.04	13.00 ± 2.15	12.98 ± 1.59
ER	0.30 ± 0.31	0.25 ± 0.19	0.27 ± 0.18	0.30 ± 0.20	0.28 ± 0.18

*PI* performance index, *SR* scanning rate, *ER* error rate.

**Figure 1:**
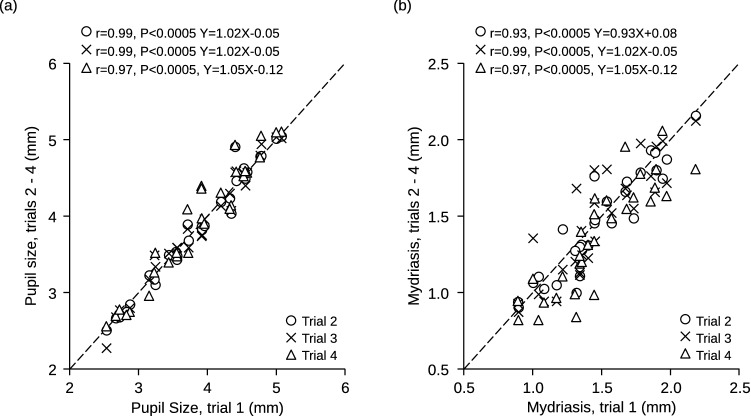
Stability of pupil size measurements in different experimental sessions. The average pupil size at rest **(a)** and the mydriasis elicited by a haptic task **(b)** have been recorded in four different experimental sessions (trials). Values relative to the second, third and fourth trial have been plotted as a function of the initial value. The corresponding coefficients of correlation and regression lines equations have been reported in both (**a**) and (**b**). Dotted lines represent equal values on X and Y axes.

**Figure 2:**
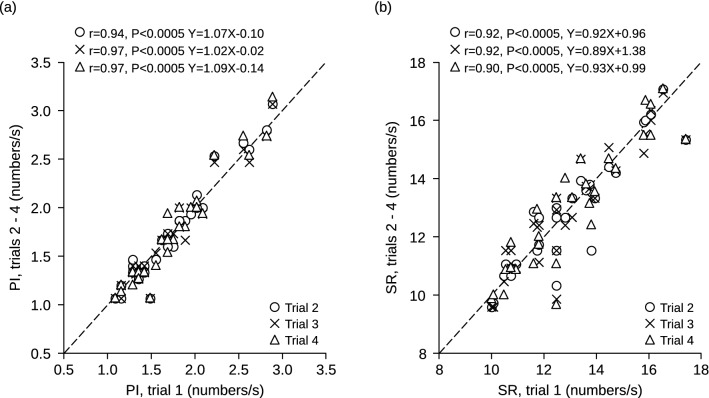
Stability of performance in different experimental sessions. The Performance Index **(a)** and the Scanning Rate **(b)** have been recorded in four different experimental sessions (trials). Values relative to the second, third and fourth trial have been plotted as a function of the initial value. The corresponding coefficients of correlation and regression lines equations have been reported in both A and B. Dotted lines represent equal values on X and Y axes.

**Figure 3:**
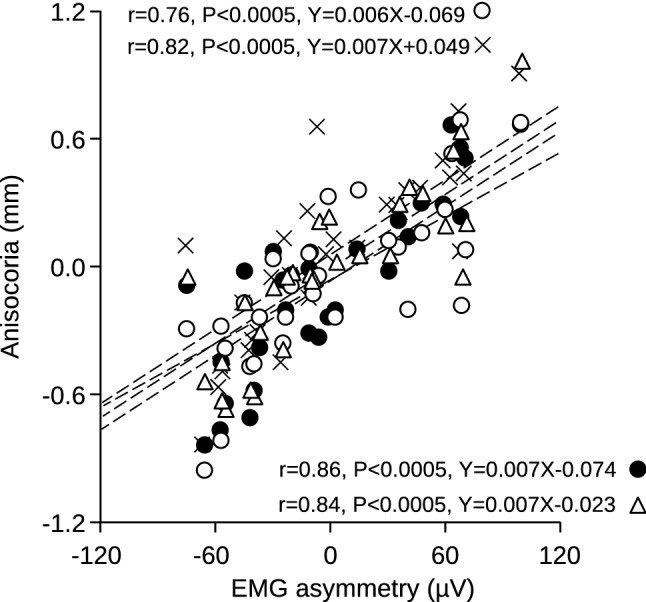
Stability of anisocoria at rest across different experimental sessions. The anisocoria (pupil size difference: left–right) at rest has been recorded in four different experimental sessions (trials) with the arches apart and the masticatory muscle relaxed. Values obtained in the different trials have been plotted as a function of the EMG asymmetry (left–right) of masseter muscles observed during clenching. Dots: trial 1, circles: trial 2, crosses: trial 3, triangles: trial 4. The dotted lines correspond to the regression lines evaluated for the different trials, whose equations are reported in the graph.

Average pupil size and task-related mydriasis values recorded in the second, third and fourth session closely mirrored those observed in the initial one (Fig. 1). The same held true for Performance Index (PI) and Scanning Rate (SR) (Fig. 2), as well as for Error Rate (ER) (not shown). Twenty out of the 30 subjects displayed in Fig. 1 showed a significant asymmetry (asymmetry index > 20%) of their masseter EMG activity during clenching. As shown in Fig. 3, this asymmetry was predictive of anisocoria when the subject kept the masseter muscle relaxed and the arches apart. Pupil size was larger on the side of higher EMG activity (hypertonic side).

In these 20 subjects the average EMG asymmetry during clenching without bite corresponded to 54.1 ± 18.5 µV. When clenching took place with bite (occlusal correction), the asymmetry was significantly reduced to 6.3 ± 6.1 µV (Bite ON vs Bite OFF, paired t-test, p < 0.0005). The change was explained by a significant drop in EMG activity at the hypertonic side (from 159.4 ± 54.1 to 131.6 ± 41.0 µV, paired t-test, p < 0.0005) and by a significant enhancement at the hypotonic side (from 105.3 ± 44.5 to 125.4 ± 39.9 µV, paired t-test, p < 0.004). Among these 20 subjects, 7 were suffering of temporomandibular joint (TMJ) disorders and/or edentulia, while the remaining 13 did not show any overt dental or mandibular problem (see “Methods”). No significant difference could be observed between the two groups in terms of EMG activity on the hypertonic and hypotonic side. Surprisingly, TMJ disorders/edentulous subjects showed a slightly lower EMG asymmetry (43 ± 16.5 µV) with respect to normal subjects (60.1 ± 17.2 µV) (t-test, p = 0.049). Bite-induced changes in EMG activity and asymmetry followed the same pattern in both groups.

### Effects of occlusal contact and orthotic placement on anisocoria and pupil size at rest

The pupil size difference (anisocoria) between the hypertonic and the hypotonic side varied across conditions (Bite ON, Bite OFF) and positions of the arches (Open, Contact), as indicated by a significant Condition × Position effect (F(2,38) = 22.23, p < 0.0005), decomposed in Table 2. In the transition between the Open and Contact position, pupil asymmetry at rest increased significantly (Bite OFF), while it decreased when the bite was interposed (Bite ON). Once the arches were opened again (Open2), the pupil asymmetry went back towards the original values, but did not recover completely. No difference could be found in the initial Open position between Bite ON and Bite OFF conditions, while in the Contact position wearing a bite lowered anisocoria by 78%. It is of interest that the same results could be obtained for both normal as well as TMJ disorders/edentulous subjects. Moreover, no differences were observed between the subpopulations of males and females, and of subjects with different occlusal dominance (i.e. right or left masseter hypertonus).

**Table 2:** Mean ± SD of anisocoria at rest and during haptic task in the different conditions (Bite ON, Bite OFF) and positions (Open 1–2, Contact) have been recorded in 20 subjects showing an asymmetric masseter EMG activity during clenching.

Condition	N = 20	Position					
	Variable	Open1	Open1 vs contact	Contact	Contact vs Open2	Open2	Open2 vs Open1
Bite OFF	Anisocoria	0.424 ± 0.278	P < 0.001	0.613 ± 0.347	P < 0.053	0.506 ± 0.317	NS
OFF vs ON		NS		P < 0.0005		P < 0.015	
Bite ON		0.425 ± 0.250	P < 0.0005	0.136 ± 0.088	P < 0.010	0.301 ± 0.289	NS
Bite OFF	Pupil size (hypertonic)	4.27 ± 0.74	P < 0.0005	4.75 ± 0.69	P < 0.0005	4.33 ± 0.72	P < 0.019
OFF vs ON		NS		P < 0.0005		P < 0.006	
Bite ON		4.30 ± 0.76	P < 0.0005	3.95 ± 0.64	P < 0.001	4.19 ± 0.81	P < 0.011
Bite OFF	Pupil size (hypotonic)	3.84 ± 0.67	P < 0.0005	4.14 ± 0.69	P < 0.0005	3.82 ± 0.70	NS
OFF vs ON		NS		P < 0.0005		P < 0.006	
Bite ON		3.88 ± 0.69	NS	3.82 ± 0.64	NS	3.89 ± 0.72	NS

Anisocoria across conditions and positions was evaluated measuring pupil size bilaterally, while the subject was not task-engaged (at rest). Pupil size per se also showed a significant Condition x Position × Side effect (F(2,38) = 22.23, p < 0.0005), decomposed in Table 2. While contact of the arches without bite increased pupil diameter at rest on both sides, contact with bite did the opposite, but only on the hypertonic side, thus indicating a particular influence of hypertonic pupil size on anisocoria changes. Wearing the bite led to a significantly smaller pupil size on both hypertonic and hypotonic side in the Contact position, while the bite was ineffective in the initial Open position. Pupil size diameters did not fully recover their initial value, when subjects opened again their arches following the contact (Open2 position, see Table 2), leading to significant differences in pupil size between Bite OFF and Bite ON conditions. These responses to changes in mandibular position and occlusal conditions were observed in normal subjects and in people affected by TMJ disorders/edentulia, independently of gender or occlusal dominance.

### Effects of occlusion and mandible position on performance and task-related mydriasis

Significant Condition × Position effects were observed for both Performance Index (PI, F(2,38) = 137.52, p < 0.0005) and Scanning Rate (SR, F(2,38) = 53.16, p < 0.00005), decomposed in Table 3. When arches changed from Open1 to Contact position, both PI and SR decreased in Bite OFF and increased in Bite ON. No difference could be found in the initial Open position between Bite ON and Bite OFF conditions, while in the Contact position wearing a bite increased PI and SR by 33% and 15%, respectively. Instead, only a position effect could be detected for Error Rate (ER, F(2,38) = 11.91, p < 0.0005), as this parameter was lower in Open2 with respect to Open1 and Contact. These responses to changes in mandibular position and occlusal condition were observed in normal subjects and in people affected by TMJ disorders/edentulia, independently of gender or occlusal dominance.

**Table 3:** Mean ± SD values of the different parameters characterizing the performance of the subject, recorded with and without Bite correction, in the open and contact position.

Condition	Variable	Position					
		Open1	Open1 vs Contact	Contact	Contact vs Open2	Open2	Open2 vs Open1
Bite OFF	Performance index	1.84 ± 0.57	P < 0.0005	1.69 ± 0.49	P < 0.0005	1.82 ± 0.57	NS
OFF vs ON		NS		P < 0.0005		P < 0.03	
Bite ON		1.85 ± 0.60	P < 0.0005	2.24 ± 0.55	P < 0.0005	1.88 ± 0.57	NS
Bite OFF	Scanning rate	13.22 ± 2.23	P < 0.0005	12.59 ± 2.12	NS	12.8 ± 2.35	P < 0.003
OFF vs ON		NS		P < 0.0005		NS	
Bite ON		13.08 ± 2.25	P < 0.0005	14.48 ± 1.96	P < 0.0005	12.79 ± 2.43	P < 0.021
Bite Average OFF/ON	ER	0.24 ± 0.15	NS	0.24 ± 0.17	P < 0.0005	0.16 ± 0.16	P < 0.001
Bite OFF	Mydriasis (average)	1.39 ± 0.32	P < 0.0005	0.78 ± 0.30	P < 0.0005	1.38 ± 0.32	NS
OFF vs ON		NS		P < 0.0005		NS	
Bite ON		1.39 ± 0.32	P < 0.0005	1.78 ± 0.43	P < 0.0005	1.45 ± 0.35	NS

*PI* performance index, *SR* scanning rate, *ER* error rate.

Similarly to PI and SR, also task-induced mydriasis showed a Condition x Position effect, decomposed in Table 3 (F(2,38) = 193.49, p < 0.0005). Changes in mydriasis mirrored those in PI and SR. Moreover, a significant Side effect (F(1,19) = 4.82, p < 0.041) showed that mydriasis was larger on the hypotonic (1.42 ± 0.38 mm), as compared to the hypertonic side (1.30 ± 0.29 mm). While the Condition x Position effect was significant in all subgroups, the side effect persisted only in the female subpopulation (F(1,12) = 9.352, p < 0.010).

### Association between changes in anisocoria, pupil size and task-related mydriasis

The changes in anisocoria at rest elicited by changing arches position and occlusal condition were positively correlated to those observed in pupil size on the hypertonic (r = 0.730, p < 0.0005, Y = 0.591X − 0.020), but not on the hypotonic side (r = − 0.114, p < 0.127).

**Figure 4:**
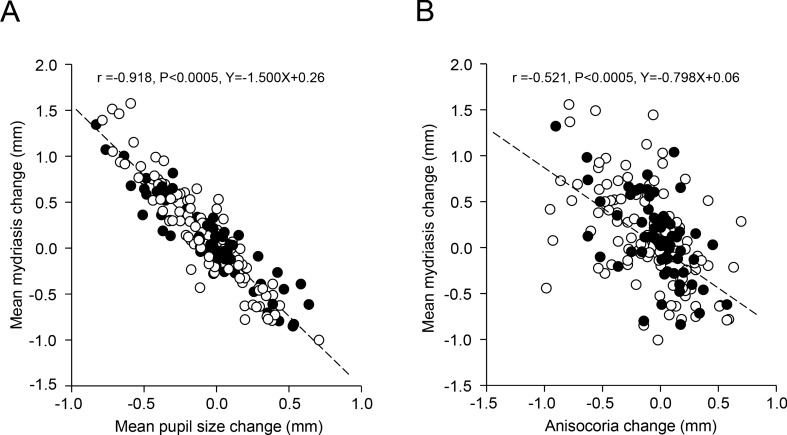
Relation between changes in task-related mydriasis, pupil size and anisocoria induced by modifying arches position and occlusal condition. Relation between changes in average task-related mydriasis elicited by modifying arches position and occlusal condition have been plotted as a function of the corresponding changes in average pupil size at rest **(A)** and anisocoria **(B)**. The dotted lines represent regression lines of all the plotted points, whose equations are displayed on the graphs. Dots and circles refer to TMJ disorders/edentulous and normal subjects, respectively.

Changes in task-related mydriasis (average of both pupils) were positively correlated with modifications in anisocoria (r = − 0.521, p < 0.0005, Y = − 0.798X + 0.06) and average pupil size at rest (r = − 0.918, p < 0.0005, Y = − 1.500X + 0.26) (Fig. 4A,B): both correlations persisted when the covariance between mydriasis and the third parameter was removed by partial correlation analysis. As shown in Fig. 4A,B, where TMJ disorders/edentulous subjects and normal subjects are represented by dots and circles, respectively, both populations were well fitted by the same regression model. This held true for subjects of different genders and occlusal dominance. A highly significant negative correlation between changes in mydriasis and pupil size at rest could be observed both on the hypertonic (r = − 0.921, p < 0.0005, Y = − 1.269X − 0.010) and on the hypotonic side (r = − 0.800, p < 0.0005, Y = − 1.399X + 0.09). Also these correlations were observed in both normal subjects and people affected by TMJ disorders/edentulia, independently of gender and occlusal dominance.

### Association between changes in performance and pupil size parameters

**Figure 5:**
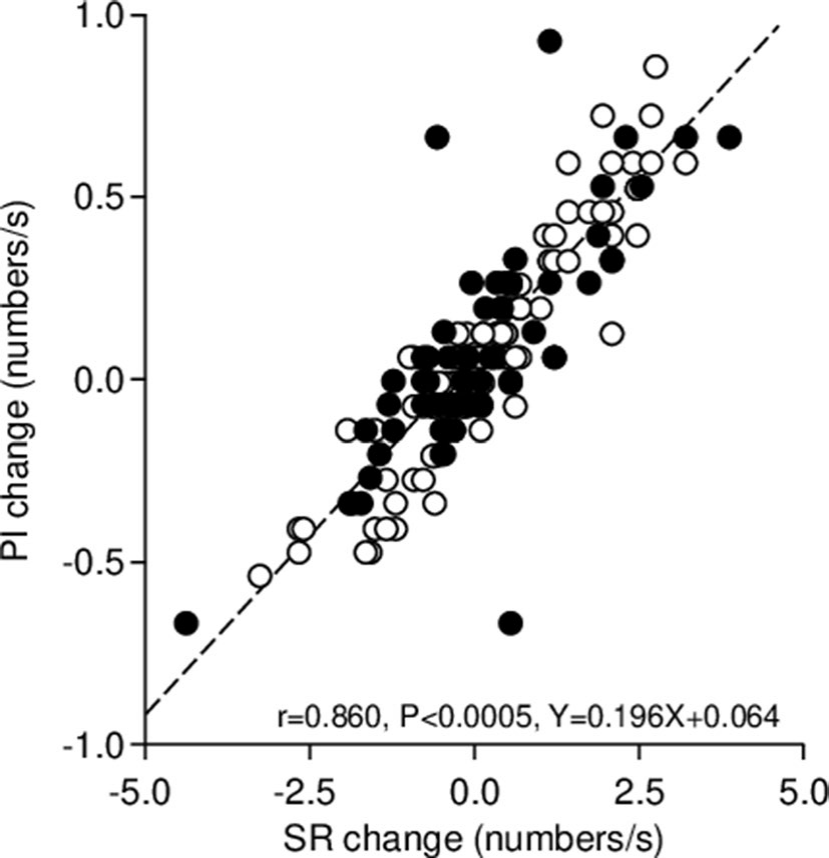
Relation between changes in the PI and the SR induced by modifying arches position and occlusal condition. The dotted line represents the regression line of all the plotted points, whose equation has been reported in the plot. Dots and circles refer to TMJ disorders/edentulous and normal subjects, respectively.

**Figure 6:**
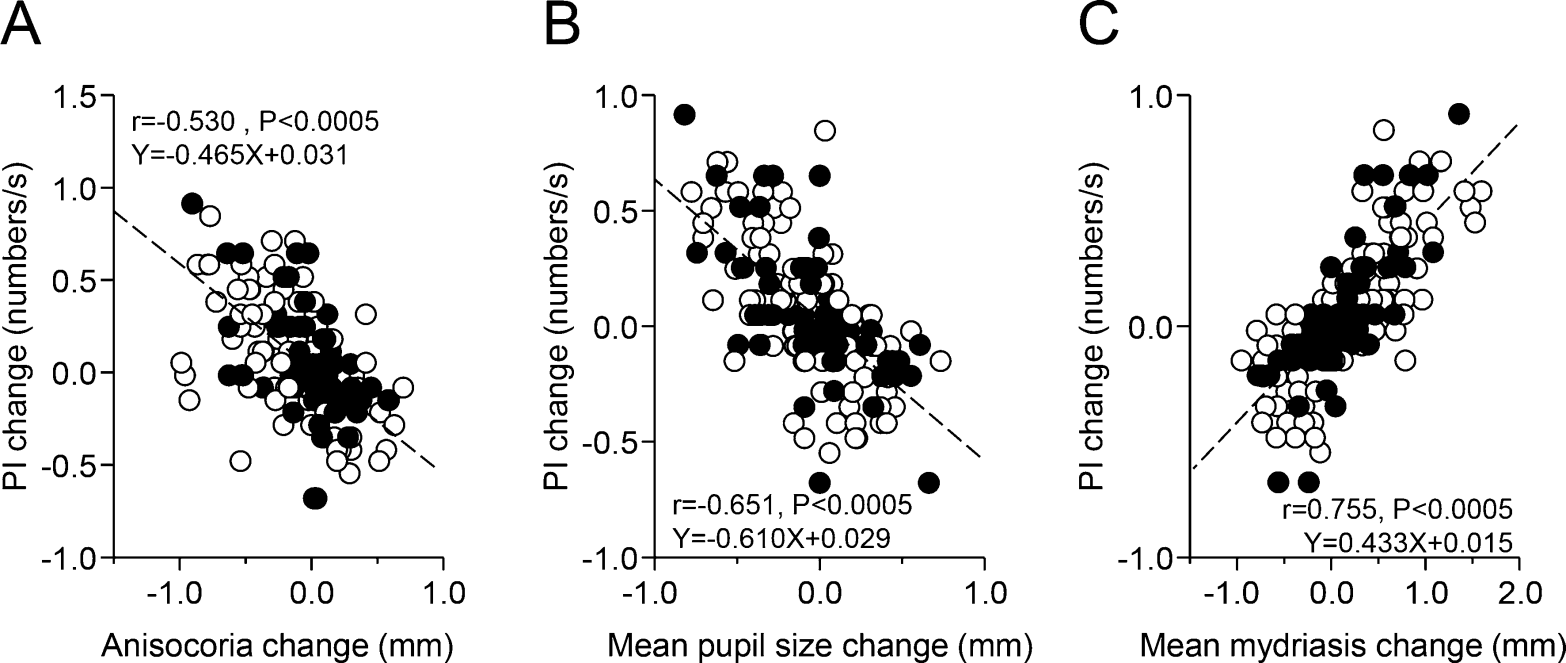
Relation between the changes in PI and those in average pupil size at rest, task-related mydriasis and anisocoria induced by modifying arches position and occlusal condition. Values of PI changes have been plotted as a function of the corresponding changes in average pupil size **(A)**, anisocoria **(B)** and task-related mydriasis **(C)**. The dotted lines represent regression lines of all the plotted points, whose equations have been reported in the graphs. Dots and circles refer to TMJ disorders/edentulous and normal subjects, respectively.

As shown in Fig. 5, changes in PI were positively correlated with those in SR (r = 0.860, p < 0.0005, Y = 0.196X + 0.064). PI and SR modifications were also associated to changes in pupil size parameters: in particular, they were negatively correlated to the corresponding changes in anisocoria (Fig. 6A, PI: r = − 0.530, p < 0.0005, Y = − 0.465X + 0.031; SR: r = − 0.449, p < 0.0005, Y = − 2.730X − 0.110) and in average pupil size at rest (Fig. 6B, PI: r = − 0.651, Y = − 0.610X + 0.029, p < 0.0005; SR: r = − 0.490, p < 0.0005, Y = − 2.016X − 0.101), while positively correlated to the average task-related mydriasis (Fig. 6C, PI: r = 0.755, p < 0.0005, Y = 0.433 + 0.015, SR: r = 0.620, p < 0.0005, Y = 1.562X − 0.163). All these correlations were present in both normal and TMJ disorders/edentolous subjects, independently of gender and occlusal dominance. Partial correlation analysis indicated that the relation of PI and SR changes with changes in pupil size at rest was generated by the covariance of pupil size changes with changes in mydriasis and anisocoria.

When the relation of PI and SR to pupil size at rest was further explored by feeding pupil size of the hypertonic and hypotonic side within the correlative model, only the hypertonic one gave a significant contribution to the variability of performance (Table 4).

**Table 4:** Multiple correlation model of the relation between PI (performance index) and SR (scanning rate) changes with pupil size changes on the hypertonic and the hypotonic side.

Variable	Coefficients	Coefficients values	Error	t	Significance
PI	Intercept	0.015	0.016	0.939	0.349
	Beta (hypertonic side pupil)	− 0.512	0.047	− 10.905	p < 0.0005
	Beta (hypotonic side pupil)	0.025	0.068	0.369	0.712
SR	Intercept	− 0.161	0.081	− 1.985	0.049
	Beta (hypertonic side pupil)	− 1.875	0.242	− 7.747	p < 0.0005
	Beta (hypotonic side pupil)	0.377	0.352	1.072	0.285

Note the low value of the beta coefficient for the hypotonic side pupil and its lack of significance.

### Aim II

#### Effects of unilateral trigeminal neurectomy on gene expression

**Figure 7:**
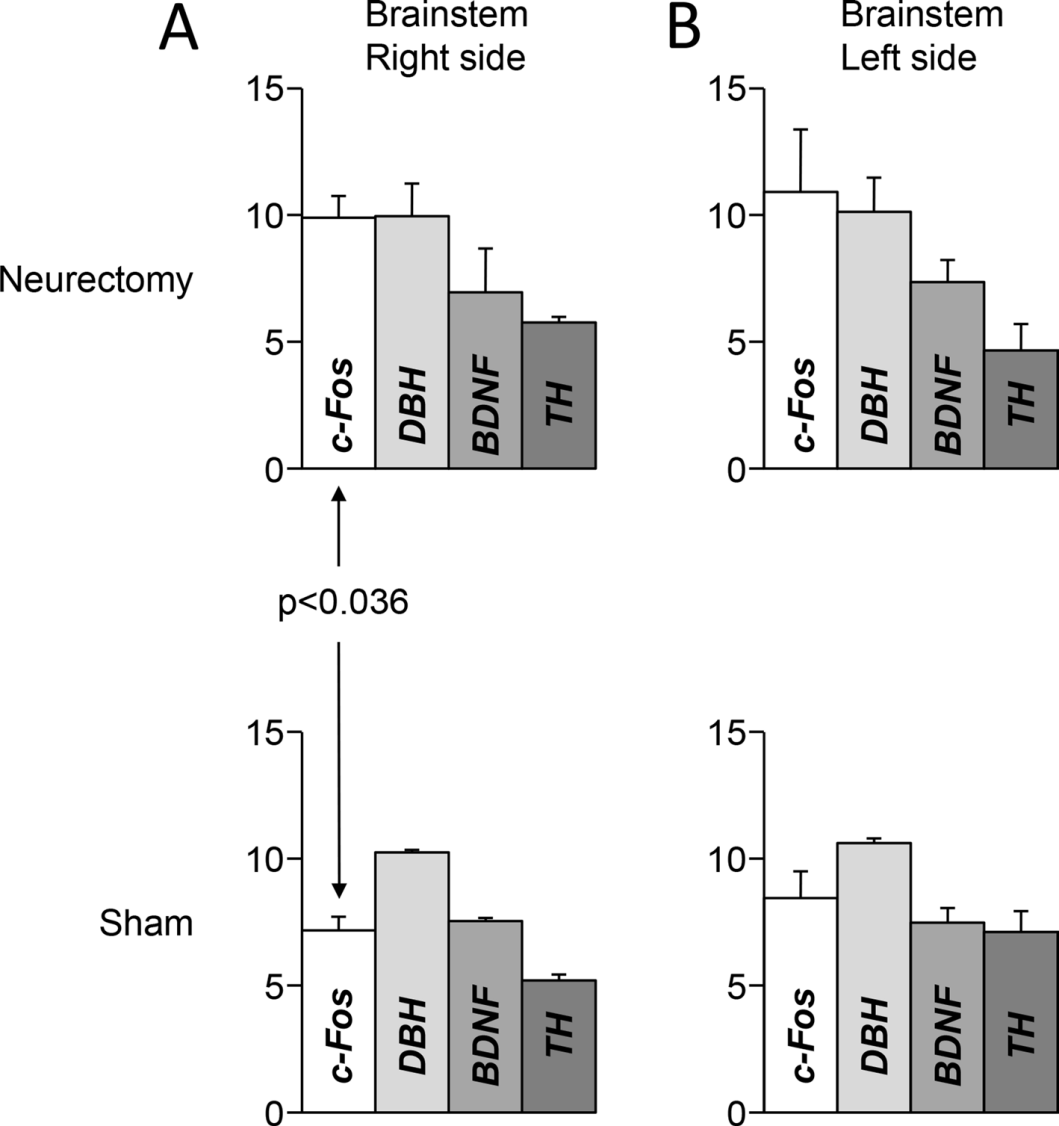
Expression levels of BDH, TH, BDNF and c-FOS within the brainstem in trigeminal neurectomy and sham preparations. The height of the white columns represents average ΔCT values evaluated with respect to ACTB (housekeeping gene). Error bars correspond to standard errors.

Within the brainstem of Wistar rats, unilateral trigeminal neurectomy decreased c-Fos expression level on the contralateral (intact) side with respect to control values, as indicated by the higher ΔCT values (see Fig. 7). On the other hand, DBH, TH and BDNF gene expression levels were not affected on the lesioned side. In this structure, no significant asymmetry in the expression of the genes investigated was found either in control or in neurectomized animals.

**Figure 8:**
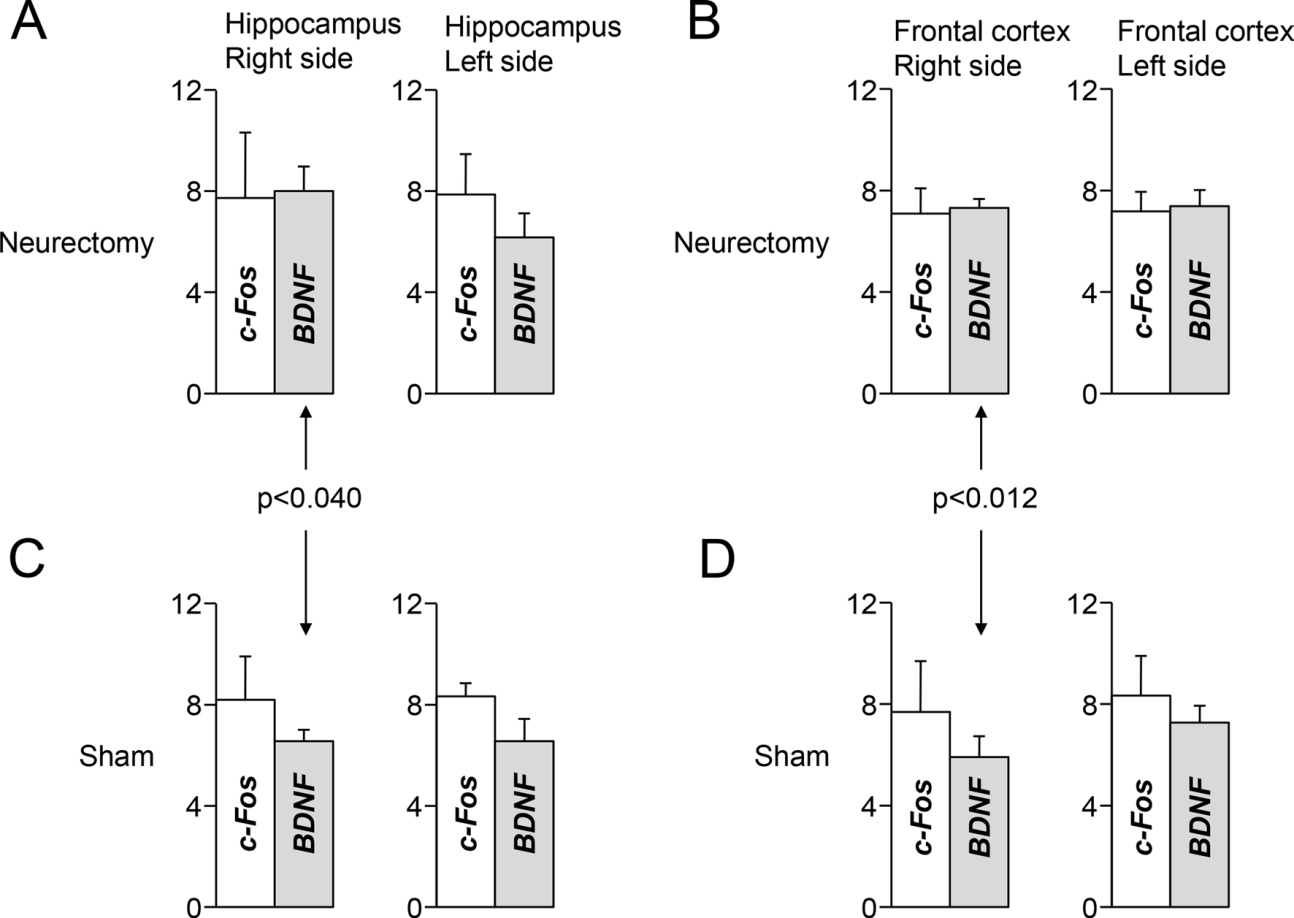
Expression levels of BDNF and c-Fos within the hippocampus and the frontal cortex of trigeminal neurectomy and sham preparations. The height of the white columns represents average ΔCT values evaluated with respect to ACTB (housekeeping gene). Error bars correspond to standard errors. **(A,C)** Hippocampus. **(B,D)** Frontal cortex. **(A,B)** Neurectomy. **(C,D)** Sham.

At both hippocampal (Fig. 8A,C) and frontal cortical (Fig. 8B,D) level, c-Fos expression did not reveal any asymmetry and its levels were not affected by neurectomy, neither on the lesioned, nor on the intact side. As to the BDNF gene expression, this was reduced on the intact (but not on the lesioned side), both in the hippocampus (Fig. 8A,C) and in the frontal cortex (Fig. 8B,D). In the hippocampus the large reduction in BDNF gene expression on the intact side led to a significant asymmetry (Fig. 8A, upper row), while this was not the case in the frontal cortex (Fig. 8B, upper row).

#### Effects of malocclusion on gene expression

Induction of unilateral (left side) malocclusion by grinding molar teeth did not significantly modify, at brainstem level, the expression of genes coding for DBH, c-Fos and BDNF, quantified by the corresponding ΔCT values, with respect to control animals. This held true for both the intact and the treated side. Similarly, no changes could be observed at hippocampal and frontal cortical levels in the expression of c-Fos and BDNF-coding genes, on both the intact as well as the treated side.

Moreover, no significant differences could be observed in both control and malocclusion groups between left and right side for all the ΔCT values analysed. Given the lack of significance, these data have not been shown in the text.

Malocclusion did not modify animal body weight.

## Discussion

### Aim I

#### Occlusal unbalance and pupil size at rest

It is established that asymmetric activity of elevator muscles during clenching is predictive of an asymmetry in pupil size (anisocoria), when the teeth are in contact without muscle effort, the side of higher EMG activity during clenching corresponding to the side of the larger pupil size^7,8^. In this paper we systematically show that, in subjects affected by an unbalance of masseter EMG activity during clenching, anisocoria can be observed also at rest, with the dental arches apart and the muscles relaxed. This highlights a tonic dimension of the occlusal unbalance observed during the phasic activity of clenching. It is likely that in these subjects the LC activity at rest is tonically asymmetric. We hypothesize that this tonic asymmetry might arise from an unbalance in the trigeminal afferent input at rest and/or in the central trigeminal pathways to the LC^32^. This hypothesis is consistent with (a) the neuroanatomical link between trigeminal afferents and the LC^32^, (b) the strong covariation existing between LC activity and pupil size^34,35^, (c) the observation that the correction of the EMG asymmetry greatly reduced also the anisocoria^7,8^. It has to be stressed that the presence of a masticatory imbalance was not dependent upon a pathological dental and/or TMJ condition, since most of our asymmetric subjects were normal.

### Pupil size and performance

In the present study the position of the arches (in contact or slightly apart) and the occlusal contact condition (Bite ON, Bite OFF) modified pupil size, task-related mydriasis and cognitive performance. In particular, when closing the arches in Bite ON, a decrease in average pupil size was observed, while both mydriasis and performance increased.

It is known that LC activity during task is tightly coupled to cognitive performance: failure to increase the LC discharge leads to poor task performance in monkey^40^, while, in humans, performance is positively correlated to pupil size during the task and task-related mydriasis^63,64^, a readout of LC activation induced by the task. Since, in the present experiments, a positive correlation was observed between trigeminal-induced changes in task-related mydriasis and performance in the matrices test (see Fig. 6C), it is likely that changes in mandible position and occlusal condition had modified LC activity and, as a consequence, cognitive performance.

In monkeys, both performance and task-related mydriasis show a peculiar dependence upon the LC activity observed at rest^40^. When the LC activity is low, performance and mydriasis are enhanced by an increased LC activity. However, above a given background level any further increase of LC activity will lead to a reduction in performance. A similar, inverted U-shaped relation has been described in humans^65^ between sustained attention and pupil size at rest, a proxy of LC activity. Other studies, possibly performed in conditions of high level of alertness, and therefore high LC activity at rest, have shown that changes in performance and task-related mydriasis were inversely related to the corresponding changes in pupil size at rest^63,66–68^. Our results are consistent with the these latter observations as, in subjects affected by masseter EMG asymmetry, changes in pupil size at rest, particularly on the hypertonic side, negatively correlated with cognitive performance.

It can be argued that, in the present experiments, subjects showed a high resting level of LC activation, likely due to the discomfort of having the head restrained by the pupilometer and simultaneously fixating the light spot displayed by the instrument for performing pupil size evaluations.

### Anisocoria and performance

When the arches were closed in Bite ON, the increase in performance was associated to a decreased anisocoria, due to a decrease in pupil size on the hypertonic side, without major changes on the hypotonic one. Diameters of both pupils increased when the bite was not interposed between the arches during contact, leading to an increased anisocoria as compared to the Open position. It is of interest that the changes in performance and task-related mydriasis were negatively correlated to those in anisocoria. The correlation with anisocoria persisted when the dependence of performance upon task-related mydriasis was removed by partial correlation analysis.

These results underline that the performance does not depend solely upon the task-related enhancement of LC activity, but also on the asymmetry of its resting activity between the two sides: the higher the imbalance, the worst being the performance. Since the correlation between performance/mydriasis and anisocoria was similar in both normal and TMJ disorders/edentulous subjects, we may conclude that the cognitive changes occur upstream of the trigeminal imbalance, independently of its origin.

These observations are consistent with the results of animal experiments showing that, in the cat, the effect of a unilateral asymmetric brain lesion can be removed by a second symmetric lesion on the other hemisphere^30^, as well as with the observation that asymmetrical brain irradiation in humans, which induces an asymmetry in brain activity (Dr. U. Faraguna, personal communication), leads to a reduced performance with respect to control subjects^69^.

### Velocity and precision in the matrices test

The trigeminal-induced changes in performance can be interpreted within the conceptual framework of speed-accuracy tradeoff, often referred to as Fitt’s law^70^: given a limited amount of cognitive resources, they tend to be preferentially allocated towards either velocity or precision.Violating this general law, trigeminal manipulations modified the scanning velocity without corresponding modifications in the number of errors, consistently with previous observations on chewing-induced changes in cognitive performance utilising the matrices test^6^. The improvement in performance despite constant error rate could be attributed to a allostatic response leading to a widespread increase in LC-induced norepinephrine release during the task^71^. This would enhance the signal-to-noise ratio of cortical and thalamic neurons^72^ leading, in turn, to an increased efficiency of sensory coding^73^ and to a better performance of those networks involved in the cognitive process^74^.

### Closing the arches with or without bite: trigeminal effects on pupil size and anisocoria

In the absence of a direct recording of trigeminal afferent activity during positional and occlusal modifications, the receptors involved in the observed phenomena remain unknown. However, the present data suggest that in subjects with an EMG asymmetry during clenching (a) an asymmetric trigeminal input is present also when the muscles are relaxed, possibly arising from muscle spindle/temporomandibular joint receptors and (b) the input asymmetry is strengthened during contact of the arches, without the interposition of the bite, i.e. with imbalanced occlusion, possibly as a result of the recruitment of periodontal receptors.

Moreover, trigeminal asymmetry and the subsequent anisocoria are reduced when the arches come into contact through the bite. This is possibly due to a reduction of afferent activity on the hypertonic side, where the pupil size is significantly reduced. While it is reasonable to assume that a basal asymmetry of trigeminal afferents activity is reduced by a correct contact of the arches, it is less clear why the associated stimulation of periodontal receptors decreases pupil size. It may well be that the establishment of a correct occlusal contact removes a somehow stressing condition arising from an incorrect mandible position. Stress may increase the discharge of lateral vestibular nuclear neurons^75^, enhancing fusimotor drive^76^. During a correct occlusal contact a reduced discharge of muscle spindle afferents may counteract the effects of periodontal afferents recruitment on LC neurons. Finally, an imbalanced occlusion could be associated to a higher attentive effort, a condition of enhanced fusimotor drive^77^. fMRI studies of brain activation during finger movements performed with an imbalanced occlusion are in agreement with this hypothesis^78^.

#### Aim II

### Changes in gene expression following the induction of trigeminal asymmetries

At variance with acute human experiments of Aim I, animal experiments of Aim II failed to show clear evidence of chronic trigeminal influences on the central noradrenergic system. In fact, no change in the brainstem level of the norepinephrine-synthesizing enzymes DBH could be observed following two and half months of malocclusion, neither on the intact nor on the treated side. Consistently, the expression of c-Fos and BDNF genes was not modified at brainstem, hippocampal and frontal cortical levels. It has been shown that a similarly induced unilateral malocclusion led to signs of masticatory muscles fibres and spindles suffering, fifteen days after the intervention^79,80^. Given the effects of trigeminal activity and trigeminal-carried neurotrophic factors at brainstem level^32^, changes in brainstem DBH expression could be expected^57–59^.

It has to be pointed out, however, that, in the present study, only the most superficial layer of the enamel was grinded and the molar cusps were not completely removed, at variance with previous studies^79,80^. A more aggressive enamel removal could lead to a greater distance between the teeth surfaces of the opposing arches, resulting in a more severe masticatory dysfunction. The same consideration may also explain the lack of a decrease in c-Fos and BDNF-coding gene expression at hippocampal level, which instead occurs following a bilateral resection of molar cusps^18,21,81^.

Similarly to malocclusion, also unilateral trigeminal neurectomy failed in modifying the brainstem expression levels of the DBH and TH. It cannot be excluded that the effects of malocclusion and neurectomy on brainstem structures fell beyond the temporal and spatial resolution of the proposed molecular approach. Indeed, introducing more time points in the experimental design and a more localized detection of protein transcription could further clarify the mechanisms involved. In the neurectomy preparation, however, a significant decrease in c-FOS expression was observed on the “intact” side of the brainstem, coupled to an ipsilateral decrease of BDNF expression in the hippocampus and in the frontal cortex. These observations expand the understanding of the spectrum of trigeminal effects on brain functions and show that even minor lesions (with respect to massive molar extraction) may modify in the long run (60 days) important signals affecting brain plasticity.

An increase in c-Fos expression has been observed 9–14 days following ipsilateral infraorbital nerve section/ligature within the spinal trigeminal nucleus^82,83^. This increase has been attributed either to an injury discharge of the severed fibres or to their uptake of neurotrophic substances released at the injury level. However no changes were reported 42 days following inferior alveolar nerve ligature^84^.

In the present experiments, c-Fos was lower than control values on the side contralateral to inferior alveolar nerve section; this finding could be related to a low activity^85^ within brainstem structures contralateral to the trigeminal section. Such a lower activity could be explained by taking into account that trigeminal neurectomy removes both dental nociceptors and periodontal receptors, whose net effect in blunting the discharge of elevator motoneurons during oromotor activities^86,87^ is well established. Unilateral removal of this sensory feedback might have favoured the development of asymmetric motor patterns, with an enhanced activation on the lesioned side and a reduced activation on the intact side, thus explaining the decrease in c-Fos expression at this level. Data on subjects wearing fixed and removable implant-supported dentures are in agreement with this hypothesis^88^.

Does the decrease of c-Fos expression at brainstem level observed on the intact size reflect corresponding changes in the activity of the LC? At a first glance, this hypothesis seems unlikely, since, in the present experiments, no modifications were observed in the expression of genes coding for the activity-dependent norepinephrine synthesizing enzymes.

The sampled brain region, however, encompasses several noradrenergic cell groups^89^ and, so far, trigeminal influences have been systematically documented only impinging on the LC. It is therefore possible that changes in the expression of LC genes coding for TH and DBH have been obscured by pooling together RNAs from neurons belonging to different noradrenergic cell groups. In this respect, the inclusion of a large brainstem portion within the sample collected for mRNA analysis—imposed by technical reasons—represents a major limitation of the study, since it prevents the accurate localization of the observed changes in the LC. As a consequence, the modifications described in the frontal cortex and in the hippocampus could not necessarily involve the LC, but rather the classical brainstem pathways arising from the trigeminal nuclei.

However, a decrease in c-Fos occurring in the LC region of the intact side would be consistent with the decrease in BDNF gene expression observed in the ipsilateral hippocampus and frontal cortex. Norepinephrine/LC stimulation promotes the synthesis of BDNF in the hippocampus^62,90^ and a similar process could take place also in the frontal cortex. Since ascending noradrenergic projections show an ipsilateral predominance^91,92^, the decrease in BDNF gene expression in the hippocampus and in the frontal cortex elicited on the intact side by unilateral neurectomy could be explained by a decrease in the activity of LC neurons at the intact side. A local decrease of BDNF may compromise the survival of noradrenergic terminals^93^, leading to a deficient noradrenergic transmission in the cortex and in the hippocampus^91,92^.

Moreover, following peripheral deafferentation, the BDNF expression observed in the corresponding sensory cortical regions decreases after 14 days, increases after 28 days, and recovers 84 days later ^94^. These changes are different from those observed in the present experiment (decrease after 60 days), thus suggesting that different mechanisms and pathways are involved. Finally, a bilateral decrease of BDNF levels in the prefrontal cortex and in the hippocampus is described also in a mouse model of depression^95^. Notably, clinical depression is accompanied by an impairment of noradrenergic transmission and shows a strong correlation with the number of spared teeth in adults and elders^96–98^. These findings further emphasize the intimate LC-mediated relation existing between trigeminal system and cognitive functions.

## Conclusions

The results obtained in acute conditions in humans indicate that changes in occlusal condition and mandible position modify the performance through changes in LC activation. In fact, performance changes were strongly correlated to those in task-related mydriasis, which can be considered as a proxy of LC activation during task. Finally, the negative correlation between modifications in performance and anisocoria at rest indicates that trigeminal-induced tonic asymmetries in LC discharge are detrimental for performance. Conversely, occlusal balancing restores cognitive performance and this is associated, on the hypertonic side, with a reduction in pupil size, a proxy of LC activity. These observations underline that occlusal balancing may represent a tool for rescuing a background afferent imbalance, improving subjective performance and may be exploited within both training and rehabilitative programs.

Signs of a chronic asymmetry in the activity of the noradrenergic system were also provided in the trigeminal neurectomy group, both at hippocampal and frontal cortical level, where BDNF values were reduced on the intact side, thus suggesting a depressed central noradrenergic activity at this level. This finding could be due to removal of inhibitory influences from periodontal receptors and nociceptors on elevator motoneurons of the lesioned side, enhancing the masticatory effort on this side and reducing the use of the intact side during oromotor activity.

Further experiments are needed in order to provide more convincing evidence of the LC involvement in chronic trigeminal effects on the brain: in this respect, an assay of protein levels directly in LC would be important for more specifically localizing activity and trophic changes in this region.

## Methods

All methods were carried out in accordance with relevant guidelines and regulations and all experimental protocols were approved by the Ethical Committee of the Pisa University and by the Italian Ministry of Health.

### Aim I

#### Subjects

Thirty subjects of both genders (age 36.3 ± 12.5, 18–55 years, 15 females) were enrolled for a preliminary screening of EMG asymmetry during clenching, while assessing stability of pupil size and evaluating performance.

Only those subjects with an EMG asymmetry greater than 20% (n = 20, age 35.2 ± 12.6, 20–54 years, 13 females) were enrolled in the experimental study. All participants signed an informed consent, approved by the Ethical Committee of the Pisa University (Unipi Bioethical Committee No: 12–2019). All subjects were right-handed and none of them was affected by metabolic, endocrine, neurological or psychiatric diseases. Among those enrolled in the preliminary study, 13 subjects had full dentition and did not complain any masticatory dysfunctions; 5 suffered loss of 1–4 molar teeth, while 3 showed signs of temporomandibular joint (TMJ) dysfunction consisting in a click as soon as the mandible started lowering and/or in pain when it reached the extreme opening position. One subject was both edentulous and showed signs of TMJ dysfunction.

#### Preliminary study

The preliminary study consisted of four sessions separated by 2–3 days. During the first session we performed the following experimental steps: (1) left and right masseter EMG activity recording during clenching; (2) pupil size measurement at rest and during performance of a haptic task (TanGram), with the arches slightly apart, in the normal resting position (Open position); (3) score in a cognitive task based on the Spinnler-Tognoni numeric matrices test^99^; in this task subjects had to scan three numeric matrices made of ten lines and ten columns, with the goal of retrieving and tick with a pencil as many target numbers (indicated above each matrix) as possible^6^.

From these data the following parameters were evaluated off-line:

(a) the increase in pupil size during task (task-related mydriasis);

(b) the difference in pupil size between the two eyes (anisocoria);

(c) the Performance Index (PI), the Scanning Rate (SR) and the Error Rate (ER) obtained in the matrices test, defined as it follows:

PI = target numbers retrieved in 15 s/15

SR = target + non target numbers scanned in 15 s/15

ER = (missed target numbers + non-target numbers wrongly underlined)/15.

Steps 2 and 3 were repeated three times, 2–3 days apart.

### Experimental study

Those subjects affected by an EMG asymmetry during clenching underwent two further sessions:

In Session 1, a dental impression (imprint) was manufactured, that stabilized the arches in a mandible position (myocentric occlusion) where clenching led to a symmetric masseter EMG activity. The symmetric myocentric occlusion was assessed when masticatory muscles had been completely relaxed after 15 min of transcutaneous, bilateral stimulation of trigeminal motor branches as described in detail elsewhere^7^. Based on this dental impression, a cusp bite was designed so to constrain the mandible position in symmetric myocentric occlusion. The patients were invited to wear such bite continuously for two weeks and all reported full compliance.

Session 2 begins with repetition of steps 2 and 3 performed in the preliminary session. The subjects, without bite in their mouth (Bite OFF) had to keep the arches apart (Open1). Once performance and pupil size data were collected, measurements were repeated two more times: first while subjects kept the arches in contact (Contact) and then once more in the initial position (Open2).

Then, the subjects had to wait about one hour before repeating the whole sequence (Open1—Contact—Open2) after Bite wearing (Bite ON).

In order to minimise learning processes associated to task repetition, target numbers varied in their position within each line in the successive tests.

### Trigeminal stimulation

Bipolar stimulation of trigeminal mandibular branches was performed by couples of electrodes (surface 1600 mm^2^) placed on the incisura sigmoidea and on the submental region of both sides through two independent I.A.C.E.R stimulators (Martellago, Venice, Italy), which delivered biphasic (cathodal/anodal) current pulses (0.54 ms, 21–25 mA). Left and right current intensity was adjusted until symmetric EMG responses were observed. Frequency of stimulation of 0.618 Hz and 40 Hz led to contraction/relaxation of masseters and to tonic contraction of lowering muscles, resulting in small amplitude mandibular movements (1 mm).

### Haptic task

In this task, subject’s head was restrained by the pupillometer, which prevented visual control. He/she had to place within a Tangram puzzle the parallelogram-shaped piece which had been previously removed, by using the right, dominant hand. Pupil size measurement was taken when the subject began the exploration of puzzle surface.

### Data acquisition

Pupil size was measured by a corneal topographer-pupillographer (MOD i02, with chin support, CSO, Florence, Italy), in constant artificial lighting of 40 lx (photopic condition). During pupil size measurement the instrument displayed a fixation light spot to the subject. The instrument, endowed with a CCD camera sensor (working distance: 56 mm) allowed to monitor and store on disk the iris image, with an acquisition time of 33 ms. Measurements were performed for both eyes.

Masseter EMG activity was recorded by Duo-trode surface Ag/AgCl electrodes (interelectrode distance 19.5 mm, MyoTronics, Seattle, WA, USA). Details about electrode placement have been given in a previous study^8^.

EMG activity was sampled at 720 Hz by a K6-I MyoTronics system, high-pass (cutoff frequency 15 Hz) and notch (50 Hz) filtered, full-wave rectified and displayed on the instruments monitor, together with the mean value of the EMG bursts produced during clenching.

### Statistical analysis

The effects of bite on the EMG activity during clenching and the corresponding EMG difference (hypertonic-hypotonic) were assessed by paired t-test. The effects of dental contact and bite on the pupil size and on task-related mydriasis was assessed by a 2 Condition (Bite ON, Bite OFF) × 3 Position (Open1, Contact, Open2) × 2 Side (hypertonic, hypotonic) repeated measures ANOVA. A 2 Condition × 3 Position ANOVA was applied in order to analyse the changes in the pupil size difference at rest, as well as in performance-related variables. These analyses were also separately performed for males-females subgroups, normal-edentulous/TMJ dysfunction subjects and right hypertonic-left hypertonic subjects. The Greenhause-Geisser ε correction was applied when appropriate. Finally, the changes elicited in pupil size and performance parameters by modifying arches position and occlusal condition were evaluated as differences:

(a) between any position and the previous ones (Contact-Open1, Open2-Contact, Open2-Open1) for both conditions (Bite ON, Bite OFF) and

(b) between Bite ON and Bite OFF condition for each of the 3 positions (Open1, Contact, Open2). The correlations between changes in pupil size and performance parameters were assessed by linear regression analysis. Statistical Package for Social Sciences (IBM Corp. Released 2011. IBM SPSS Statistics for Windows, Version 20.0. Armonk, NY: IBM Corp. https://www.ibm.com/support/pages/downloading-ibm-spss-statistics-20) was used for the analysis and significance was set at p < 0.05.

### Aim II

This study was approved by the Animal Ethical Committee of the University of Pisa and by the Italian Ministry of Health (Authorization No: 469/2015-PR).

Twenty Wistar Han male rats (Envigo RMS Srl S. Pietro al Natisone—Udine—Italy) were used in the study and randomly assigned to 2 experimental procedures aimed at inducing a trigeminal asymmetry: (1) Unilateral molar cusps grinding (malocclusion); (2) Inferior alveolar nerve resection.

#### Unilateral malocclusion

Five control and five treated animals (age 15–18 months) underwent deep anesthesia (Sodium pentobarbital, i.p., 60 mg/kg); the left molar teeth cusps were removed (for about 0.4–0.5 mm) in treated animal by gently grinding with a micro drill, as described elsewhere^79,80^. After 75 days animals were sacrificed, their brains collected by sectioning the medulla at the level of the caudal border of the cerebellum (Bregma -15) and promptly immersed in RNAlater (Qiagen, Hilden, Germany) according to the manufacturer instructions. Total mRNA was independently extracted from the left and right brainstem, the frontal cortex and the hippocampus by using the RNeasy Mini Kit (Qiagen, Hilden, Germany) and reverse-transcripted by using the Quantitect Reverse Transcription Kit (Qiagen, Hilden, Germany). Relative expression of target genes (Table 5) was obtained by means of Quantinova SYBR Green PCR Kit (Qiagen, Hilden, Germany) using Beta-Actin (ACTB) (Forward: 5′-AGCGTGGCTACAGCTTCACC-3′; Reverse: 5′-AAGTCTAGGGCAACATAGCACAGC-3′)^100^ and Glyceraldehyde 3-Phosphate Dehydrogenase (GADPH) (Forward: 5′-TGCCCCCATGTTTGTGATG-3′; Reverse: 5′-GCTGACAATCTTGAGGGAGTTGT-3′)^100^ as reference genes.

**Table 5:** Inferior alveolar nerve resection: primers used in gene expression analysis.

Anatomical Area	Gene	Group (s)	Primers	Reference
Brainstem	c-Fos	Malocclusion	Forward: 5′-GATGTTCTCGGGTTTCAACG-3’	Mazurek et al.^102^
		Trigeminal neurectomy	Reverse: 5′-CGCAAAAGTCCTGTGTGTTG-3’	
	BDNF	Malocclusion	Forward: 5′-GCCCAACGAAGAAAACCATAAG-3’	Shen et al.^103^
		Trigeminal neurectomy	Reverse: 5′-GTTTGCGGCATCCAGGTAATT-3’	
	DBH	Malocclusion	Forward: 5′-AGCCCCTTCCCTTACCACA-3’	Pfeil et al.^104^
		Trigeminal neurectomy	Reverse: 5′-TGCGTTCTCCATCTCACCTC-3’	
	TH	Trigeminal neurectomy	Forward: 5′-TGTGTCCGAGAGCTTCAATG-3’	Roessner et al.^105^
			Reverse: 5′-GTCAATGGCCAGTGTGTACG-3’	
Hippocampus	c-Fos	Malocclusion	Forward: 5′-GATGTTCTCGGGTTTCAACG-3’	Mazurek et al.^102^
		Trigeminal neurectomy	Reverse: 5′-CGCAAAAGTCCTGTGTGTTG-3’	
	BDNF	Malocclusion	Forward: 5′-GCCCAACGAAGAAAACCATAAG-3’	Shen et al.^103^
		Trigeminal neurectomy	Reverse: 5′-GTTTGCGGCATCCAGGTAATT-3’	
Frontal cortex	c-Fos	Malocclusion	Forward: 5′-GATGTTCTCGGGTTTCAACG-3’	Mazurek et al.^102^
		Trigeminal neurectomy	Reverse: 5′-CGCAAAAGTCCTGTGTGTTG-3’	
	BDNF	Malocclusion	Forward: 5′-GCCCAACGAAGAAAACCATAAG-3’	Shen et al.^103^
		Trigeminal neurectomy	Reverse: 5′-GTTTGCGGCATCCAGGTAATT-3’	

Target genes: c-Fos, brain-derived neurotrophic factor (BDNF), dopamine beta-hydroxylase (DBH), tyrosine hydroxylase (TH).

### Inferior alveolar nerve resection

Five control animals and five treated animals (age 17–20 months) underwent deep anesthesia (mix of dexmedetomidine 200 mcg/kg, ketamine 20 mg/kg, midazolam 1 mg/kg and fentanyl 5 mcg/kg, i.m); treated animals underwent the resection of the left inferior alveolar nerve^101^. In particular, the bony canal was drilled and 2 mm of nerve extent were removed. Control animals underwent only bony canal exposure (Sham). After 60 days, animals were sacrificed and their brains collected and processed as mentioned above. Table 5 details the investigated target genes; ACTB and GADPH were used as housekeeping genes.

### Animal health

The animals were individually housed in cages in a room with a 12 h light/dark cycle and constant temperature (21 °C). Food and water were available ad libitum and their condition was continuously monitored. Only animals in apparent good health were included in the study. Before treatment their average weight was 573 ± 62. This value was lower than the reference one observed for obese Wistar rats of comparable age^106^.

Following the treatment, animal weight was periodically monitored before sacrifice: a six time (pre 0–14 days, post 1–4 days, post 5–8 days, post 9–12 days, post 20–40 days, post 4–60 days) repeated measures ANOVA did not show any significant Time effect in the control and experimental animals of both groups.

### Statistical analysis

∆CT values from each animal were used for comparisons. For each target gene, intra-animal (left vs right) and intergroup (same side: control vs treated) values were compared using the paired and independent t-test, respectively. The Statistical Package for Social Sciences (IBM Corp. Released 2011. IBM SPSS Statistics for Windows, Version 20.0. Armonk, NY: IBM Corp. https://www.ibm.com/support/pages/downloading-ibm-spss-statistics-20) was used for the analysis and significance was set at p < 0.05. If not specified otherwise the reported descriptive statistics corresponds to Mean ± Standard Deviation.

### Aim I and II: age comparison

The present study reports data obtained in humans (Aim I) and rats (Aim II). Data in humans bearing a trigeminal asymmetry have been collected in a sample ranging from 20 to 54 years. The age of Wistar rats at the time of treatment ranged from 15 to 20 months. This range corresponds to 37- 49 years in humans^107^. At sacrifice, animal age ranged from 19 to 24 months, equivalent to 48–60 years in humans. Therefore, the animal age partially overlapped with that of human subjects.
